# Dental Phenotype in Jalili Syndrome Due to a c.1312 dupC Homozygous Mutation in the *CNNM4* Gene

**DOI:** 10.1371/journal.pone.0078529

**Published:** 2013-10-23

**Authors:** Hans U. Luder, Christina Gerth-Kahlert, Silke Ostertag-Benzinger, Daniel F. Schorderet

**Affiliations:** 1 Institute of Oral Biology, Center of Dental Medicine, University of Zurich, Zurich, Switzerland; 2 Department of Ophthalmology, University Hospital, Zurich, Zurich, Switzerland; 3 Clinic of Orthodontics and Pedodontics, Center of Dental Medicine, University of Zurich, Zurich, Switzerland; 4 Institut de Recherche en Ophtalmologie, Sion, Switzerland; 5 Department of Ophthalmology, University of Lausanne, Lausanne, Switzerland; 6 Faculty of Life Sciences, Ecole Polytechnique Fédérale de Lausanne, Lausanne, Switzerland; University of North Carolina at Chapel Hill, United States of America

## Abstract

Jalili syndrome denotes a recessively inherited combination of an eye disease (cone-rod dystrophy) and a dental disorder (amelogenesis imperfecta), which is caused by mutations in the *CNNM4* gene. Whereas the ophthalmic consequences of these mutations have been studied comprehensively, the dental phenotype has obtained less attention. A defective transport of magnesium ions by the photoreceptors of the retina is assumed to account for the progressive visual impairment. Since magnesium is also incorporated in the mineral of dental hard tissues, we hypothesized that magnesium concentrations in defective enamel resulting from mutations in *CNNM4* would be abnormal, if a similar deficiency of magnesium transport also accounted for the amelogenesis imperfecta. Thus, a detailed analysis of the dental hard tissues was performed in two boys of Kosovan origin affected by Jalili syndrome. Retinal dystrophy of the patients was diagnosed by a comprehensive eye examination and full-field electroretinography. A mutational analysis revealed a c.1312 dupC homozygous mutation in *CNNM4*, a genetic defect which had already been identified in other Kosovan families and putatively results in loss-of-function of the protein. The evaluation of six primary teeth using light and scanning electron microscopy as well as energy-dispersive X-ray spectroscopy showed that dental enamel was thin and deficient in mineral, suggesting a hypoplastic/hypomineralized type of amelogenesis imperfecta. The reduced mineral density of enamel was accompanied by decreased amounts of calcium, but significantly elevated levels of magnesium. In dentin, however, a similar mineral deficiency was associated with reduced magnesium and normal calcium levels. It is concluded that the c.1312 dupC mutation of *CNNM4* results in mineralization defects of both enamel and dentin, which are associated with significantly abnormal magnesium concentrations. Thus, we could not disprove the hypothesis that a disrupted magnesium transport is involved in the development of the dental abnormalities observed in Jalili syndrome.

## Introduction

The term Jalili syndrome (OMIM 217080) was proposed by Parry et al. [1] for a recessively inherited combination of cone-rod dystrophy (CRD) and amelogenesis imperfecta (AI), which was observed in a large Arab kindred from the Gaza strip and first described by Jalili and Smith in 1988 [2]. Since then, cases from nine additional families (one also from the Gaza strip, two from Kosovo, and the rest from various countries) have been reported. In these patients, ocular symptoms resulting from the retinal dystrophy became apparent early in life, usually in childhood. They included photophobia, reduced vision, and nystagmus and sometimes progressed to nyctalopia [1–6].

In comparison with the ocular consequences of the disease, the dental manifestations have obtained less attention. The enamel dysplasia which invariably accompanied the retinal dystrophy has been mostly characterized as hypoplastic/hypomineralized type AI [3–5]. Only Parry et al. [1] claimed that the enamel of affected individuals appeared similar to that seen in hypomaturation type AI. In several families, consistent taurodontism of permanent molars was noticed [1], but Michaelides et al. [3] also observed obliteration of the pulp chambers.

After two studies [3,7] had found an association of CRD/AI with a locus on chromosome 2q11, two further investigations [1,4] simultaneously identified the disease-causing mutations in the ancient conserved domain protein 4 (CNNM4). Missense, termination, deletion, and single base duplication mutations were observed, which occurred in conserved regions of the protein and were assumed to result in loss-of-function. The CNNM4 protein is believed to be involved in the transport of metal ions, most likely magnesium (Mg) [8], which in the retina is essential for proper function of the photoreceptors. Since *CNNM4* has been shown to be expressed in the enamel and dentin forming cells, the ameloblasts and odontoblasts, respectively, it has been speculated that the mineral deficiency observed in affected dental enamel also results from a disturbance of Mg transport [1,4]. Our hypothesis was that if this speculation were correct, Mg concentrations in defective enamel associated with *CNNM4* mutations would be abnormal. Therefore, the aim of the present investigation was to examine in detail the mineralization density and elemental composition of enamel and dentin in patients affected by Jalili syndrome. We demonstrate that softening and discoloration of the dysplastic teeth are indeed associated with significantly abnormal Mg concentrations in both dental hard tissues.

## Materials and Methods

### Patients

Two boys from reportedly non-consanguineous Kosovan parents were referred to specialized institutions by the family pediatrician, because both siblings had poor vision and yellow-brown primary teeth. A clinical genetic evaluation at the age of 5 years 2 months and 2 years 1 month arrived at the tentative diagnosis of CRD/AI syndrome. After this diagnosis had been received, more detailed ophthalmic and dental examinations as well as mutational analyses of both the boys and the parents were envisaged. They were approved by the institutional ethics committee (Ethics Committee of the Canton of Zurich, Switzerland). Written informed consent of the parents was obtained for further processing of extracted teeth and for the isolation of DNA from peripheral blood.

### Ophthalmic examinations

Patients and parents received a comprehensive eye examination including dilated fundus assessment. Visual acuity tests in the boys were chosen depending on their age. Full-field electroretinography (ERG) was performed in accordance to the ISCEV standard in the older of the two brothers.

### Dental examinations

The elder boy was first seen by a pediatric dentist at the age of 4 years 11 months for an intraoral and radiographic examination. During the subsequent period of observation, three primary maxillary incisors, a primary maxillary molar, and a primary mandibular incisor were extracted at relatively advanced stages of root resorption. The first visit of the younger boy took place at the age of 2 years 11 months. Only 9 months later, the primary mandibular right second molar had to be extracted under general anesthesia because of carious decay and advanced apical inflammation. Both parents were examined intraorally and using panoramic radiographs.

### Microscopic analysis

All six primary teeth removed from the patients were processed for a microscopic investigation. Six discarded and completely anonymized primary teeth which had been extracted for orthodontic reasons, were healthy, and corresponded in tooth type to the specimens from the patients served as controls. Undecalcified longitudinal ground sections were prepared as described previously [9]. The microscopic analysis comprised light microscopy, backscattered scanning electron microscopy (BSE), and energy-dispersive X-ray spectroscopy (EDS). BSE imaging served for measuring the thickness of dental enamel. In the dentin, mantle dentin as well as two levels of circumpulpal dentin distant and close to the pulp cavity were assessed separately. Thresholding of backscattered signal intensities was used for discriminating intertubular and peritubular dentin. This allowed determining the percentage fractions of the two components. In addition, mineral densities of enamel and dentin were estimated based on BSE signal intensities. These measurements were made at two locations in each individual tooth, both as averages in circumscribed areas and along a 10 pixel-wide straight line from the crown surface to the pulp chamber [10]. The EDS analysis was chosen for assessing the elemental composition of the hard tissues, because it is not destructive and allows quantitative estimates of percentage concentrations of constituents with an accuracy and precision of about ±1-2% [11]. EDS spectra were recorded at the locations where mineral densities had been determined. A Si(Li) detector and counting times of 100 sec, a high tension of 7 kV and a working distance of 23 mm were used. Spectra were evaluated quantitatively with the proprietary INCA energy software (Oxford Instruments).

### Statistical analysis

Estimates of mineral densities and concentrations of elements obtained from the patients’ and control teeth were statistically compared using analyses of variance with repeated measurements (SPSS v20; IBM). A factor “disease” was included to distinguish affected and healthy teeth, while the site of evaluation and the level of the dentin sample within individual teeth were considered repeated measurements.

### Mutational analyses

Peripheral blood was taken from all family members. Genomic DNA was isolated from leukocytes by organic extraction. All exons of *CNNM4* were PCR amplified and sequenced as described in Polok et al. [4].

## Results

### Ophthalmic findings

The parents observed photophobia, reduced vision, strabismus and jiggling eyes since very early childhood. Ophthalmic findings were similar in both brothers. Reduced vision of 20/200 in both eyes was assessed without progression over the observed time period (ages 3 to 5 in the younger and ages 4 to 8 in the older brother). Cycloplegic retinoscopy revealed high hyperopia of +8.0 Dpt to +9.0 Dpt. Orthoptic evaluation demonstrated fully accommodative esotropia. Horizontal pendular-jerk nystagmus was seen in the younger boy. Ophthalmoscopy revealed macular changes (Bull’s eye maculopathy) and a pale optic disc in both patients. ERG recording showed reduced rod responses and non-recordable cone responses at age 8 years in the older brother.

Neither the mother nor the father complained about ocular problems, and upon clinical examination, their vision proved excellent. A dilated eye assessment performed in both of them did not reveal any abnormalities. In view of the unremarkable clinical findings and lack of any symptoms, no electrophysiological tests were made.

### Dental findings

**Figure 1:**
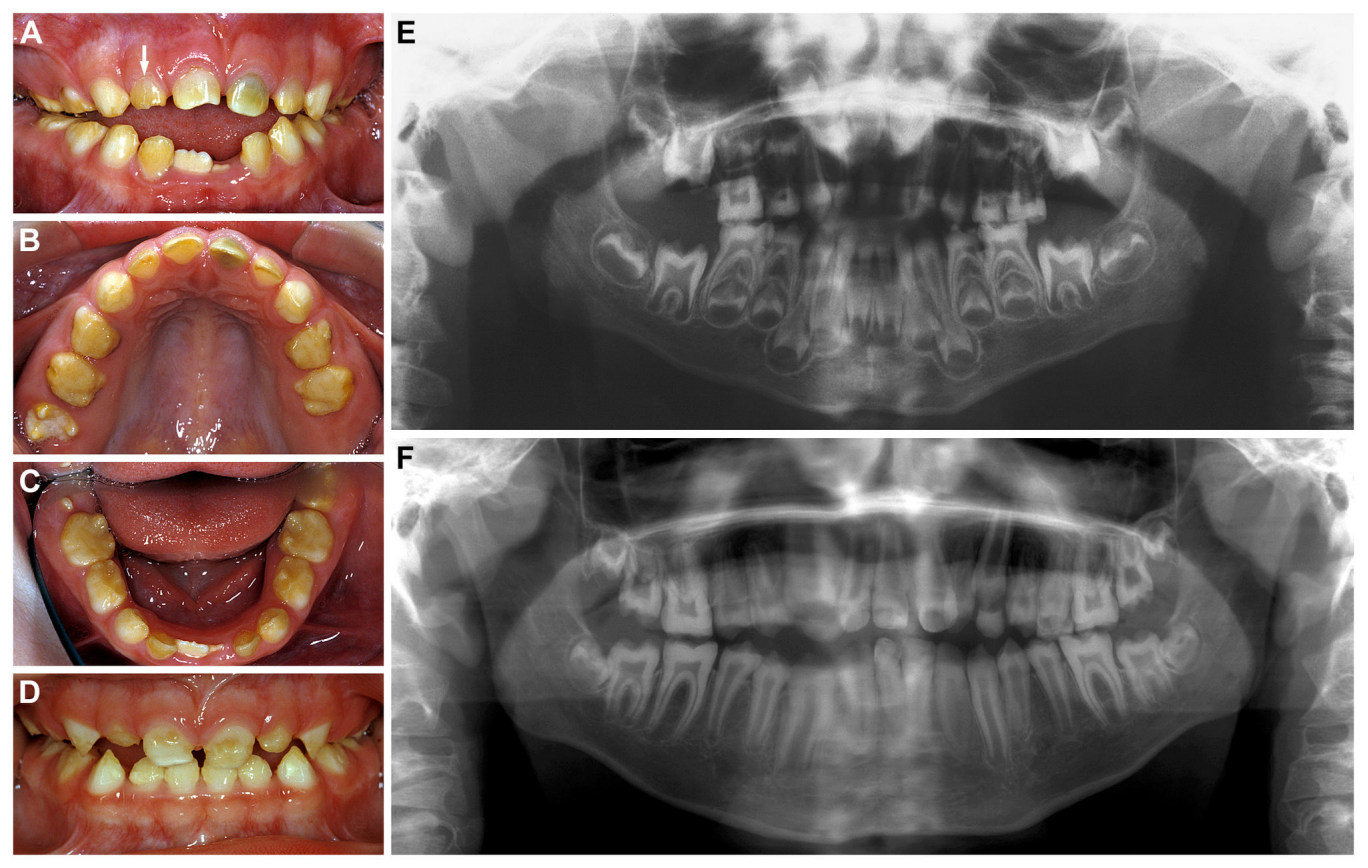
Appearance of the teeth in the two affected brothers. (**A**-**C**): Yellow-brown discolored primary teeth of the elder boy at the age of 6 years; white arrow points at the maxillary incisor shown microscopically in Figure 2. (**D**): Groove-shaped enamel hypoplasias in primary teeth of the younger boy at the age of 2 years 11 months. (**E**, **F**): Dental enamel cannot be distinguished from dentin in the panoramic radiographs taken from the elder boy at 4 years 7 months (**E**) and 10 years 6 months of age (**F**); large pulp cavities seen during eruption of permanent teeth (**E**) become normal, after teeth are in occlusion (**F**).

All primary teeth of both boys were opaque yellow-brown (Figure 1-A-D), and those of the younger brother also showed groove-shaped enamel hypoplasias (Figure 1-D). Radiographically, enamel could not be distinguished from dentin (Figure 1-E, F). At the time of eruption, the pulp chambers of the permanent molars appeared rather large (Figure 1-E), but once teeth had arrived in occlusion, the size of the pulp cavities became inconspicuous (Figure 1-F).

In contrast to those of the children, the dentitions of both parents did not reveal any even subclinical abnormalities, although some teeth were missing. Except for a few fillings, crowns were inconspicuous in shape and exhibited age-appropriate colors. Radiographically, enamel could readily be distinguished from dentin and was normal in thickness. Tooth roots were also inconspicuous and, in particular, did not reveal taurodontism. In the father, there were a few carious lesions which, however, did not necessitate extractions enabling a histopathological examination.

**Figure 2:**
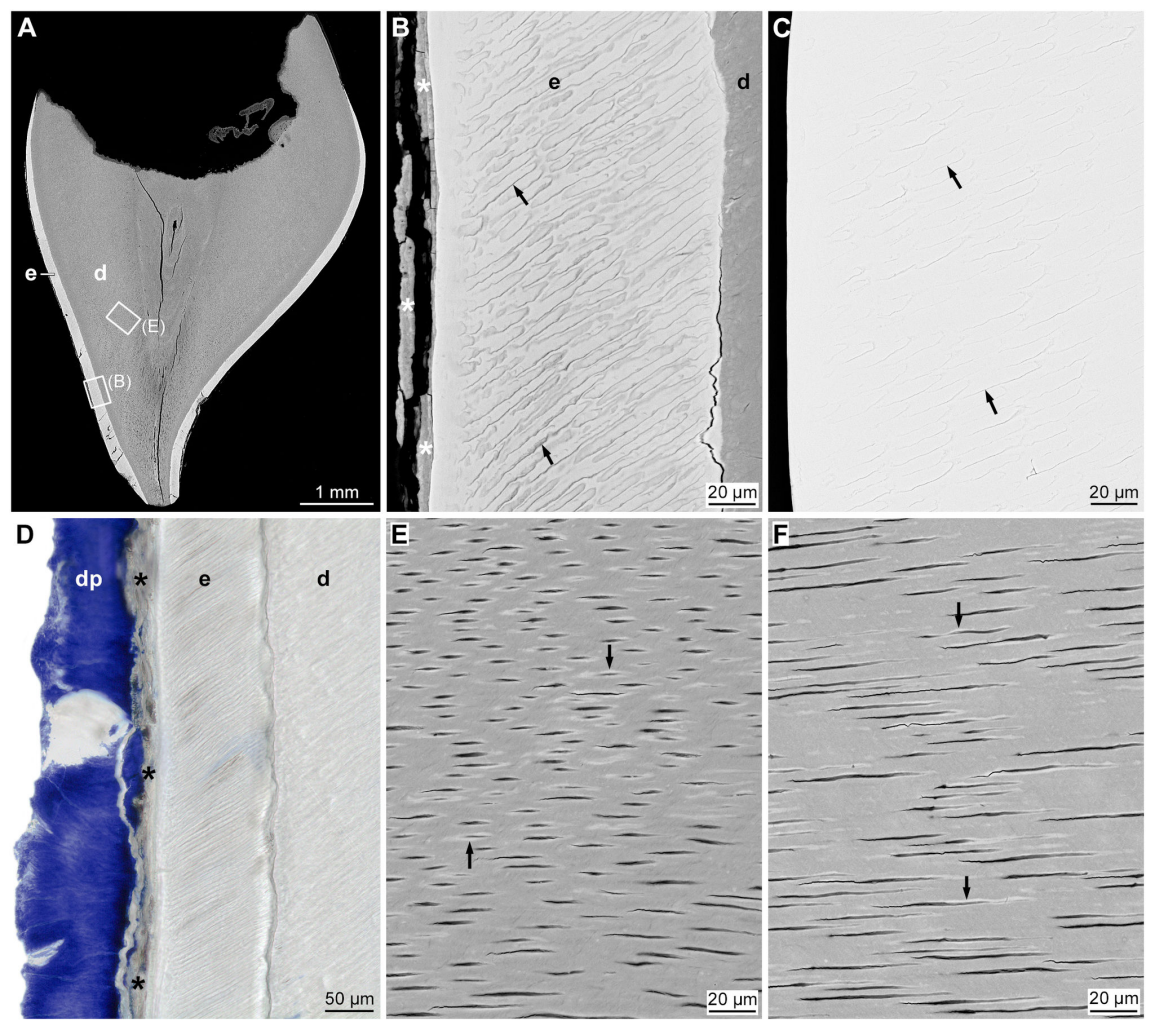
Appearance of affected primary teeth in calibrated backscattered electron images and a light micrograph. (**A**): Overview of maxillary incisor crown disclosing thin enamel (e) covering the dentin (d); rectangles mark the details shown in B and E. (**B**, **C**): In comparison to healthy enamel (**C**), enamel (e) from the affected tooth (**B**) exhibits wide, dark-gray borders between the prisms (arrows) and is covered by thin, mineralized deposits, presumably coronal cementum (asterisks). (**D**): These deposits (asterisks), in turn, are covered by dental plaque (dp) stained intensely with toluidine blue. (**E**, **F**): Dentin from affected (**E**) and healthy (**F**) teeth exhibits similar proportions of light (densely mineralized) peritubular dentin (arrows) separating the broader intertubular dentin from the dentinal tubules, but both types of dentin appear darker (less mineralized) in affected teeth (**E**). Original magnifications (**A**): 80x, (**D**): 200x, (**B**, **C**, **E**, **F**): 3000x.

**Figure 3:**
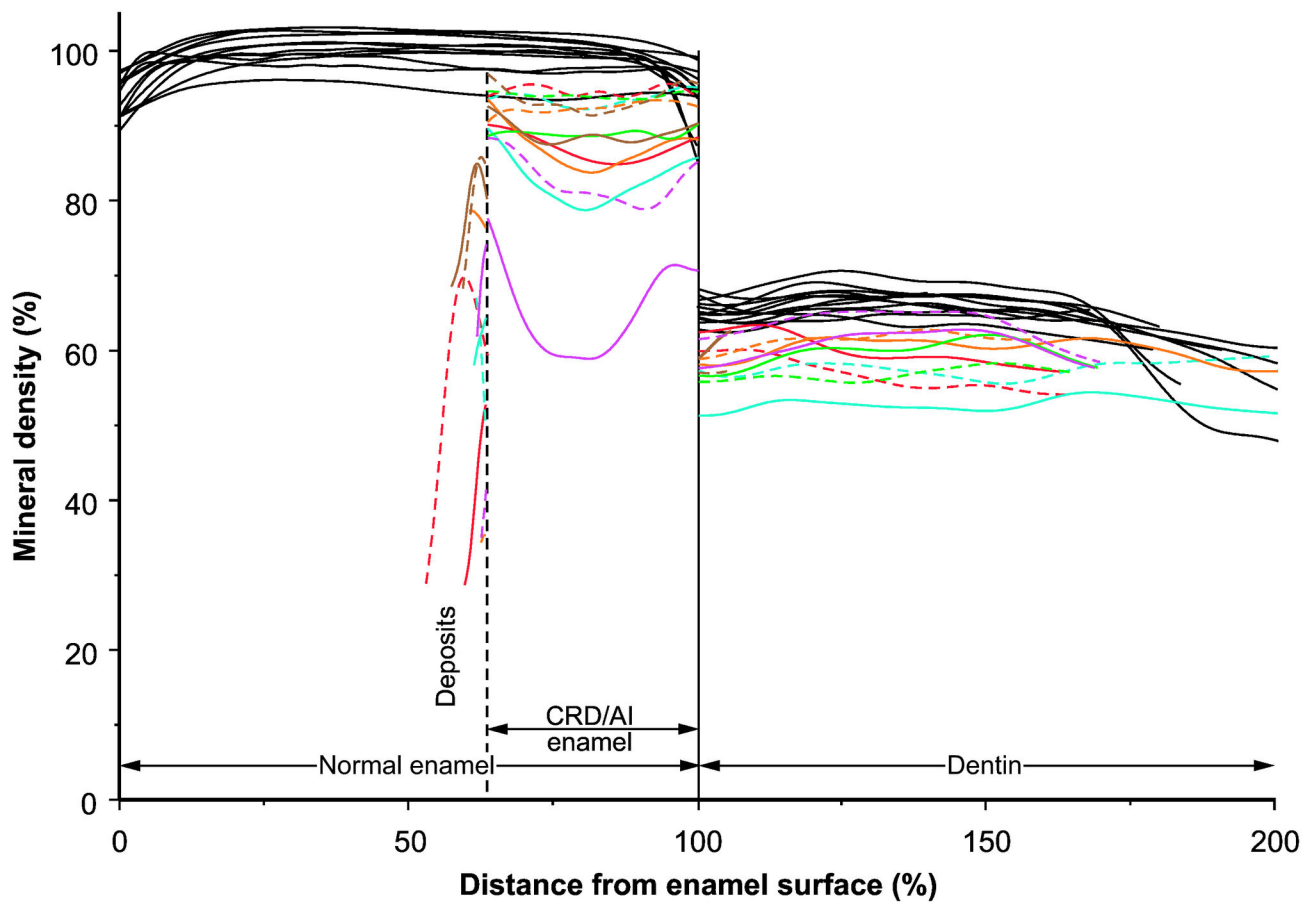
Mineral Density across Enamel and Dentin of Control and Affected Teeth. The distance scale is normalized with respect to the average thickness of healthy enamel and dentin; as a result, the surface of a normal tooth corresponds to 0%, the dentin-enamel-junction to 100%, and the dentin-pulp-border to 200%. The thickness of affected (CRD/AI) enamel corresponds to the average across all specimens examined; mineral densities to the left of affected enamel concern deposits on the surface. For the affected teeth, estimates from the two sites of each tooth are plotted as dotted and solid lines of the same color; note the large variation between and within individual CRD/AI teeth, which was significantly (p = 0.03) higher than in control specimens.

Microscopically, enamel existed in all teeth extracted from the patients, but was significantly reduced in thickness (Figure 2-A, B; Table 1) to values between 83% and 6% of control measurements. In addition, the mineral density of enamel was also significantly reduced (Table 1), on the average by about 10-15%. However, the mineral deficiency was not uniform, but varied markedly between and within affected teeth and seemed to be particularly prominent in the middle parts of the enamel layer (Figure 3). Higher magnifications (Figure 2-B) further showed that the borders between prisms were much wider and mineralized less densely than in healthy enamel (Figure 2-C). In agreement with the results from mineral density estimation, the concentration of Ca in the enamel was significantly reduced, although the Ca/P molar ratio was significantly elevated. Also the Mg/P molar ratio was significantly increased, but this was largely due to an abnormally high Mg content (Table 1). Large parts of the enamel surface along the entire crowns of affected teeth were covered with thin, mineralized deposits (Figure 2-B). They exhibited a layered structure and a degree of mineralization between those of enamel and dentin. Invariably, the deposits were covered by a thick layer of dental plaque (Figure 2-D).

**Table 1:** Structural properties and composition of enamel and dentin from healthy control teeth and teeth affected by Jalili syndrome (CRD-AI).

**Hard Tissue**	**Measurement**	**Healthy teeth (N = 6)**		**CRD-AI teeth (N = 6)**		
		**Mean**	**Range**	**Mean**	**Range**	**Significance of factor disease**
Enamel	Thickness (µm)	401	164-834	156	91-308	p = 0.04
	Mineral density (%)	99.0	98.5-100.1	87.6	75.2-91.0	p = 0.02
	Concentration Ca (wt %)	28.7	28.2-29.4	27.0	26.1-27.7	p = 0.006
	Molar ratio Ca/P	1.36	1.35-1.37	1.41	1.36-1.44	p = 0.01
	Concentration Mg (wt %)	0.2	0.15-0.2	0.3	0.25-0.35	p = 0.02
	Molar ratio Mg/P	0.016	0.014-0.017	0.025	0.02-0.031	p = 0.009
Dentin^1^	Concentration Ca (wt %)	21.9	21.5-22.6	24.2	18.4-27.3	ns^3^,^6^
	Molar ratio Ca/P	1.32	1.32-1.32	1.37	1.31-1.41	p = 0.05
	Concentration Mg (wt %)	0.65	0.64-0.66	0.35	0.25-0.45	p < 0.001^4^
	Molar ratio Mg/P	0.065	0.064-0.066	0.034	0.024-0.042	p < 0.001
Peritubular dentin	Fraction (%)^2^ in mantle dentin	1.1	0.3-1.7	0.7	0.3-1.3	ns^3,4^
	Fraction (%)^2^ in peripheral CPD	6.7	3.4-9.1	6.3	2.8-14.5	ns^3,4^
	Fraction (%)^2^ in inner CPD	5.2	2.1-7.6	5.6	3.0-8.9	ns^3,4^
	Mineral density (%)	78.1	76.1-80.9	73.8	71.4-79.5	p = 0.002^5^
Intertubular dentin	Fraction (%)^2^ in mantle dentin	98.2	97.4-99.1	98.8	98.2-99.3	ns^3,4^
	Fraction (%)^2^ in peripheral CPD	89.0	86.7-91.8	89.2	81.2-94.2	ns^3,4^
	Fraction (%)^2^ in inner CPD	85.8	85.0-86.9	89.1	86.6-92.7	ns^3,4^
	Mineral density (%) in mantle dentin	64.5	64.1-65.2	56.1	51.8-58.3	p = 0.001^4^
	Mineral density (%) in peripheral CPD	66.7	65.5-68.0	60.4	56.5-63.1	p = 0.001^4^
	Mineral density (%) in inner CPD	67.0	65.7-68.1	59.4	54.0-61.7	p = 0.001^4^
Dentinal tubules	Fraction (%)^2^ in mantle dentin	0.8	0.7-0.9	0.5	0.2-1.1	ns^4, 6^
	Fraction (%)^2^ in peripheral CPD	4.4	3.9-4.8	4.8	2.8-7.4	ns^4, 6^
	Fraction (%)^2^ in inner CPD	9.1	7.2-11.0	5.6	2.2-7.4	ns^4, 6^

1 Spatial resolution of the EDS detector did not allow distinguishing peritubular and intertubular dentin

2 Area occupied by hard tissue (µm^2^)/100 µm^2^ of reference tooth region (CPD = circumpulpal dentin)

3 ns: p > 0.05

4 Significant difference between mantle dentin, peripheral and inner CPD: p < 0.05

5 Non-significant difference between mantle dentin, peripheral and inner CPD: p > 0.05

6 Significant interaction (p < 0.05) of factor disease and difference between mantle dentin, peripheral and inner CPD

Differences between the dentin from patients and healthy individuals were more subtle than those of enamel. Whereas the proportions of peritubular and intertubular dentin as well as the course and proportion of dentinal tubules were similar, the mineral density was significantly reduced in both types of affected dentin (Table 1; Figure 2-E, F). The mineralization deficiency was less pronounced than in enamel and seemed to concern particularly the peripheral and middle parts of the dentin (Figure 3). The Ca concentration did not differ significantly from that of control teeth, but the Ca/P molar ratio was significantly raised, and unlike in enamel, the Mg/P molar ratio was significantly reduced due to a marked reduction in the Mg content (Table 1).

### Mutational analyses

Mutation analysis of *CNNM4* revealed a c.1312dupC (p.L438Pfs9X) homozygous mutation in both affected children. The parents were heterozygous for the mutation.

## Discussion

In agreement with earlier investigations [3,5], the findings of this study showed that the c.1312 dupC homozygous mutation of *CNNM4* results in AI of the hypoplastic/hypomineralized type. Although the mineral deficiency was considerably less prominent than that determined previously [1], it was associated with a significant elevation in the Mg content of enamel. In addition, the mutation also seemed to impair the mineralization of dentin, but in contrast to enamel, Mg levels were reduced.

Investigations on the effects of genetic defects on dental enamel in humans are hampered by the fact that the ameloblasts are completely lost, when teeth erupt into the oral cavity. Similarly, the dental pulp of primary teeth at advanced stages of root resorption (such as were available for this study) is transformed to granulation tissue and no original functional odontoblasts exist any longer. For this reason, conclusions regarding the consequences of genetic mutations have to be largely based on indirect evidence. On the other hand, dental hard tissues are rather stable, because they are not subject to remodeling and turnover. Therefore, their appearance and composition, irrespective of the time of examination, correspond fairly well to the condition after initial formation.

The c.1312 dupC (p.L438Pfs9X) mutation of *CNNM4* identified in the two boys of the investigated family has also been found in other, particularly Kosovan families [1,4]. Apart from changing leucine 438 into proline, it introduces a premature stop codon and, assuming the mutated mRNA is not subjected to non-sense mediated decay, results in a markedly truncated protein, which is thought to lack its normal function related to the transport of metal ions, especially Mg. This assumption fits well to our findings obtained from the affected teeth, which exhibited a prominent mineralization deficiency associated with significant alterations in the Mg content of the dental hard tissues.

It is well established that high concentrations of Mg in mature enamel are accompanied by decreased mineral density as well as diminished Ca and P contents [12], but the role of Mg and the mechanisms regulating its levels during enamel formation are obscure. Initial mineral crystals laid down in the protein matrix during the secretory stage of enamel development apparently contain high proportions of Mg. Until the onset of enamel maturation during the second stage of amelogenesis, Mg concentrations rise even further [13], although it is not clear whether this Mg is incorporated in the lattice of the mineral crystals, bound at their surface, or associated with the matrix proteins. Concomitant with the final growth of the apatite crystals during enamel maturation, Mg levels drop sharply [13]. Considering (1) that the enamel from our patients contained elevated amounts of Mg (2), that the consequence of the identified mutation in *CNNM4* is a loss-of-function, and (3) that *CNNM4* is expressed in secretory [4] and maturation stage [1] ameloblasts, it is tempting to speculate that the defective CNNM4 protein is unable to remove Mg from the maturing enamel. As a result, residual Mg would interfere with the formation of normal hydroxyapatite [12], leading to an abnormal mineral composition (as suggested by the altered Ca/P ratio) and reduced mineral density.

An intriguing finding was the presence of mineralized deposits on large parts of the affected enamel. Although these deposits were mostly covered by dental plaque, they occurred on crown areas which are easily accessible to oral hygiene and where normally no dental calculus develops. Furthermore, unlike dental calculus, deposits were mineralized rather densely and revealed thin layers running more or less parallel to the crown surface. Therefore, they most likely constituted acellular-afibrillar (coronal) cementum. This type of cementum is believed to develop, when the reduced enamel epithelium disintegrates prematurely and allows mesenchymal cells of the dental follicle to gain access to the enamel [14]. In the teeth from the examined patients, an early impairment of function and possibly death of the enamel epithelium is indeed suggested by the reduced thickness of the enamel.

Involvement of dentin in the tooth dysplasia associated with Jalili syndrome has been suggested by Parry et al. [1] based on the consistent observation of taurodontism. This conclusion was not confirmed either by a previous [3] or the present study. Our findings rather indicate that mutations in *CNNM4* affect the mineralization of dentin. In contrast to that of enamel, however, the mineral deficiency of dentin was accompanied by a lower than normal Mg and slightly elevated Ca concentration. This could be interpreted to suggest that in the odontoblasts which also express *CNNM4* [1,4], the defect of the protein impairs the transport of Mg into the tissue. As Mg of the dentin mineral is believed to compete with Ca and to be bound to phosphate [15], the observed slightly increased Ca content would also appear to agree with this assumption.

In summary, it is concluded that the c.1312 dupC mutation of *CNNM4* leads to a premature termination of amelogenesis resulting in thin, incompletely mineralized enamel, whereas in dentin, only mineralization is disturbed. In both dental hard tissues, the mineral deficiency seems to be associated with significant alterations in the concentration of Mg. Thus, our findings do not exclude the possibility that a defective cellular transport of Mg may indeed be the common cause of both the retinal and dental disease.
